# Deprotonation by Dehydration: The Origin of Ammonium Sensing in the AmtB Channel

**DOI:** 10.1371/journal.pcbi.0030022

**Published:** 2007-02-09

**Authors:** David L Bostick, Charles L. Brooks III

**Affiliations:** 1 Department of Molecular Biology, The Scripps Research Institute, La Jolla, California, United States of America; 2 Center for Theoretical Biological Physics, University of California San Diego, La Jolla, California, United States of America; University of California San Diego, United States of America

## Abstract

The AmtB channel passively allows the transport of NH_4_^+^ across the membranes of bacteria via a “gas” NH_3_ intermediate and is related by homology (sequentially, structurally, and functionally) to many forms of Rh protein (both erythroid and nonerythroid) found in animals and humans. New structural information on this channel has inspired computational studies aimed at clarifying various aspects of NH_4_^+^ recruitment and binding in the periplasm, as well as its deprotonation. However, precise mechanisms for these events are still unknown, and, so far, explanations for subsequent NH_3_ translocation and reprotonation at the cytoplasmic end of the channel have not been rigorously addressed. We employ molecular dynamics simulations and free energy methods on a full AmtB trimer system in membrane and bathed in electrolyte. Combining the potential of mean force for NH_4_^+^/NH_3_ translocation with data from thermodynamic integration calculations allows us to find the apparent pK_a_ of NH_4_^+^ as a function of the transport axis. Our calculations reveal the specific sites at which its deprotonation (at the periplasmic end) and reprotonation (at the cytoplasmic end) occurs. Contrary to most hypotheses, which ascribe a proton-accepting role to various periplasmic or luminal residues of the channel, our results suggest that the most plausible proton donor/acceptor at either of these sites is water. Free-energetic analysis not only verifies crystallographically determined binding sites for NH_4_^+^ and NH_3_ along the transport axis, but also reveals a previously undetermined binding site for NH_4_^+^ at the cytoplasmic end of the channel. Analysis of dynamics and the free energies of all possible loading states for NH_3_ inside the channel also reveal that hydrophobic pressure and the free-energetic profile provided by the pore lumen drives this species toward the cytoplasm for protonation just before reaching the newly discovered site.

## Author Summary

Selective flow of ammonium manifests itself in a unique way in the case of the ammonium channel, AmtB, allowing it to interact closely with cytoplasmic signal transduction proteins in order to “sense” the presence of extracellular ammonium. Although it is well known that AmtB transports ammonia (NH_3_) rather than ammonium ion (NH_4_^+^), it is unclear from the channel's atomic structure exactly where and how, along its pathway toward the cytoplasm, NH_4_^+^ becomes deprotonated to form NH_3_, and reprotonated on the cytoplasmic end of the channel to form NH_4_^+^ to enter the cell. We use a combination of molecular dynamics simulation techniques to glean the thermodynamics associated with these key events in ammonium translocation. Our findings provide a novel perspective on how this family of channels indirectly controls ammonium protonation—by directly controlling its hydration. Such a perspective should lend new insight to interpretations of experimental data, and could possibly lead to new strategies in an envisioned future for the design of nanopores that can control the protonated state of permeant species.

## Introduction

The transport of (NH_4_^+^) ammonium and/or (NH_3_) ammonia (we will refer to both of these species together as Am) across biological membranes is a homeostatic necessity in both prokaryotes and eukaryotes [1]. In the case of many different plants, bacteria, and fungi, Am serves as a readily available nitrogen source for biosynthetic purposes. On the other hand, at high concentrations, it becomes cytotoxic, especially in animal cells. The family of Am transport proteins—ammonium transporters (Amt) in plants and bacteria, methylamine permeases (MEP) in yeast, and rhesus (Rh) proteins in animals—serves to facilitate the permeation of Am across the membrane. Plant [2–5] and yeast [6,7] Amt/MEPs as well as many bacterial [8–10] Amts take in Am in a membrane electrochemical potential–dependent manner in order to utilize it. In humans, the related Rh proteins are split into two groups: erythroid (RhAG, RhD, and RhCE)—expressed on the erythrocyte surface [11,12] where they perform immunogenic and structural roles, and nonerythroid (RhCG, RhBG, and RhGK)—expressed in the kidneys, liver, and testes where they aid in disposal of ammonium and regulation of pH [13,14].

Many years of study have shown that while members of the Am transporter family share homologous sequences and structures, it does not necessarily follow that they conduct Am using the same mechanism [15]. Whether particular members of the family transport Am in its ionic (NH_4_^+^) or “gas” (NH_3_) form remains a subject of debate [15]. It is also suggested that some members of the family transport H^+^ *and* NH_3_ in a coupled fashion [4,10,16]. Recently, X-ray diffraction studies [17,18] have revealed the atomic structure of the bacterial Am transporter, AmtB, from *Escherichia coli,* providing a quantum leap in our understanding of the permeation of Am in the form of NH_3_. Even though its tomato plant homolog, LeAMT1;2 *does not* appear to share the NH_3_ transport mechanism, the fact that AmtB's human (kidney) homolog *does* transport NH_3_ rather than NH_4_^+^ means that these new crystallographic structures will provide an excellent starting point for understanding the structural basis of Am transport via its “gas” intermediate [15] as it pertains to human physiology. In fact, the newly available structural information on AmtB has already spurred comparative structural and functional modeling of human Rh proteins [19].

In the membrane, AmtB exists as a homo-trimer, with each monomer consisting of a right-handed eleven-helix bundle [17,18]. The center of each monomer forms a narrow hydrophobic pore connecting cytoplasmic and periplasmic depressions (vestibules). The diffraction studies revealed a NH_4_^+^ binding site (named “Am1”[17]) on the periplasmic depression where the cation donates a hydrogen bond to the hydroxyl oxygen of a Ser residue (S219) and is putatively stabilized by cation–π interactions with aromatic residues (W148 and F107). Though the narrow hydrophobic pore (lumen) connecting the inner and outer vestibules of the X-ray structure was seen to accommodate three poorly ordered NH_3_ binding sites (named Am2, Am3, and Am4, respectively [17]), the opening from the periplasmic vestibule to the pore was apparently blocked by two stacked Phe sidechains (F107 and F215), rendering the means of NH_4_^+^ deprotonation and translocation unclear. The fact that the pore was lined by two His residues (H168 and H318) hydrogen bonded to one another led to the suggestion that NH_4_^+^ can penetrate deep into the pore up to the middle site, Am3, before undergoing His-mediated deprotonation to form NH_3_ [17]. However, no site or putative mechanism for reprotonation of NH_3_ to form a cation was found. Nor was any binding site for NH_4_^+^ found in the cytoplasmic vestibule. It was also found very unlikely that water occupies the hydrophobic pore, further supporting the notion that AmtB transports NH_3_ instead of NH_4_^+^. Since AmtB passively transports Am along an electrochemical gradient and was not seen to undergo conformational changes upon occupying different permeant species, it was deemed safe [17] to consider the family of Amts as “channels.”

The new structural knowledge of AmtB has facilitated computational studies [18,20–23] aimed at understanding aspects of Am transport through the channel. Of particular interest in these studies is an Asp residue (D160) on the periplasmic side of the channel, which is highly conserved across Amt/MEP/Rh proteins. Mutational studies have shown this residue to be functionally crucial for Amts as well as MEP and Rh proteins [24,25]. Although it was proposed that D160 likely participates in the periplasmic binding site for NH_4_^+^ [24], X-ray structures revealed that the carboxylate side chain does not participate in the Am1 binding site, is sequestered from water, and interacts closely with amide N-H groups of an outer loop (connecting helices M4 and M5), hence playing an indispensable structural role in luring NH_4_^+^ from the periplasm by forcing it to interact with outwardly directed carbonyl oxygens [17,18].

A computational free energy perturbation study by Luzhkov et al. [22] demonstrated that the charged form of D160 is stabilized by 0.3–5.1 pK_a_ units in the absence of a cation at site Am1. In the presence of a cation at Am1, the charged form of D160 was seen to be even more stable (by 9.2 pK_a_ units). Performing the alchemical mutation, D160N, in agreement with experimental mutational studies [24,25] gave rise to a loss of binding for NH_4_^+^ at site Am1, suggesting that the presence of D160 stabilizes cation binding via through-space electrostatic interactions. However, the simulations of Luzhkov did not evaluate the structural role of D160 in facilitating cation sequestration using backbone carbonyls in the M4–M5 loop as suggested by structural studies [17,18]. Such an evaluation would, necessarily, involve long simulations of the mutant channel. Thus, it is probably most accurate to say that D160 plays both an important role in stabilizing cation binding at site Am1 as well as stabilizing the M4–M5 loop for recruiting NH_4_^+^ from the bulk, and that its structural and cation-stabilizing roles likely cannot be considered separately.

Computational studies performed by Lin et al. and Nygaard et al. led to the suggestion of an entirely different role for D160 as a proton acceptor from NH_4_^+^, with either water [20] or the carbonyl oxygen of A162 [23] as an intermediary, based on qualitative inspection of molecular dynamics (MD) trajectories. The simulation studies of Luzhkov et al. and Lin et al. suggested that NH_3_ is highly favored over NH_4_^+^ at the first internal binding site, Am2, while Nygaard et al. suggested that NH_4_^+^ must bind to site Am2 *before* becoming deprotonated, as suggested by Khademi et al. [17].

The available structural data and somewhat disjoint mechanistic proposals based on computational studies lead to questions about the specific details (NH_4_^+^ recruitment, deprotonation to form NH_3_, and subsequent NH_3_ translocation and reprotonation) of Am transport through AmtB: if NH_4_^+^ loses a proton somewhere between the Am1 and Am2/Am3 binding sites, exactly where and how does the deprotonation occur? What role do the blocking Phe residues (F107 and F215) play? What role does water play in the periplasmic and cytoplasmic vestibules, and can it permeate the channel? Given that once Am exists as NH_3_ in the interior of the channel, the driving force for translocation of NH_3_ toward the cytoplasmic vestibule to become *re-*protonated is unclear. What drives this uncharged species toward the cytoplasmic vestibule during the conduction event? Why are the internal NH_3_ binding sites (Am2, Am3, and Am4) poorly ordered in the X-ray structure of AmtB, and (on a related note) can all three binding sites be occupied simultaneously? Where and by what means does NH_3_ become protonated on the cytoplasmic side of the pore? And finally, is there a cytoplasmic binding site for NH_4_^+^?

## Results/Discussion

**Figure 1:**
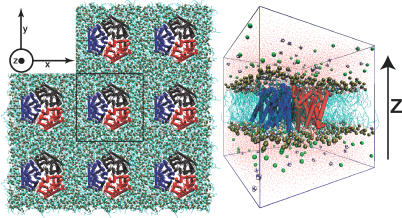
The AmtB Trimer/Membrane/Electrolyte System Setup (Left) A top-down view of the simulated system alongside several periodic images. The simulation cell boundaries are drawn in black around the central image. Lipid headgroup phosphorus and nitrogen atoms are shown as orange spheres, and lipid chains are light blue lines. The AmtB trimer is colored by monomer. (Right) A side view of the central simulation cell. Water is shown as red points, Cl^−^ ions are shown as green spheres, and NH_4_^+^ ions are shown in a space-filling representation (nitrogen is blue and hydrogens are white).

To answer these questions, we performed MD simulations on a full AmtB trimer embedded in a palmitoyloleoylphosphatidylcholine (POPC) bilayer (Figure 1) bathed in 158 ± 5 mM NH_4_Cl electrolyte solution at biological pH (see Materials and Methods). Although such a large biomembrane/electrolyte system was expected to require large equilibration times [26–29] due to slow ion-membrane adsorption, it allowed for a more large-scale view of NH_4_^+^ recruitment at the periplasmic and cytoplasmic vestibules. In addition, this system allowed us to determine ionic densities as a function of the transport (*z-*) axis from which we may derive the free energetics describing Am transport all the way from the bulk phase into the channel interior.

### Vestibular NH_4_^+^ Recruitment and Binding

**Figure 2:**
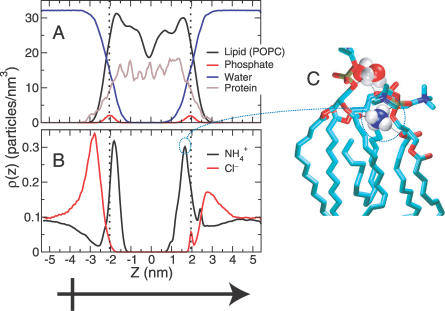
Partial Densities across the Simulation Cell Shows the system structure as a function of the transport axis, *z* (*z* = 0 coincides with the center of mass of the trimer backbone). (A) Major system components (lipid, water, and protein). The partial density of the phosphate group is shown as a reference for roughly identifying the boundaries of the membrane. A black, vertical dotted line is drawn at the position of the peak in each leaflet's phosphate density. (B) NH_4_^+^ and Cl^−^ densities. A black vertical dotted line is drawn at leaflet phosphate positions. The average concentration of NH_4_^+^ and Cl^−^ in the bulk is “measured” to be 158 ± 5 mM, based on this plot. Cl^−^ binding is enhanced on the cytoplasmic (*z* < 0) surface and inhibited on the periplasmic (*z* > 0) surface due to the “external” field created by the channel (dipole moment symbol) as seen from the Cl^−^ density maxima at ∼±2.7 nm. NH_4_^+^ binding (see density maxima at ∼±1.7 nm) is not as seriously affected, although its local concentration at ∼±2.7 nm (around the Cl^−^ binding positions) is enhanced near the periplasmic (compared with the cytoplasmic) membrane surface. (C) NH_4_^+^ binding to the membrane surface involves penetration, dehydration, and hydrogen bonding with carbonyl and ester oxygens near the glycerol backbone of lipid molecules as seen in previous work [29].

Analysis of our simulated system showed that the large dipole moment (∼2,000 Debye—see Figure S1A) of the AmtB trimer has an interesting impact on the local ionic concentration at the membrane/electrolyte interface (Figures 2, S1, and S2), which comprises a dielectric response to the protein's electric field. While NH_4_^+^ adsorption at the membrane surface was not affected significantly, Cl^−^ adsorption was. Overall, the local concentration of cation near the periplasmic vestibule (just beneath the lipid headgroup phosphate plane) was observed to be ∼16 times greater than that near the cytoplasmic vestibule. On the other hand, Cl^−^ was very unlikely to approach either vestibule, and its equilibrium concentration was ∼3 times smaller on the periplasmic side than on the cytoplasmic side (Figure S2). Hence, without any external electrochemical gradient, we see that the channel is built for recruiting cations from the periplasm at neutral pH on a mesoscopic scale. In addition we see that the channel inhibits anionic adsorption to the periplasmic membrane surface—another mechanism of enhancing cation recruitment.

**Figure 3:**
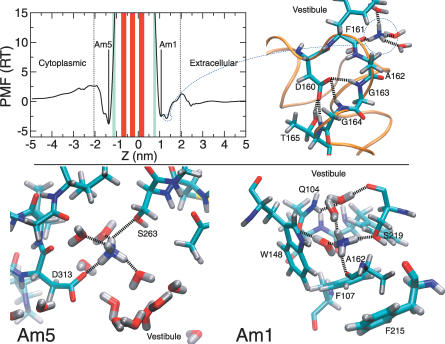
PMF for NH_4_^+^ Along the Transport Axis In units of *RT* = 2.48 kJ/mol at 298 K. The profile was shifted such that it takes on a value of zero in the bulk. The origin is the same as in Figure 2, with periplasm (*z* > 0) and cytoplasm (*z* < 0) separated by the membrane (membrane phosphate positions are marked with vertical dotted lines). Am (de)protonation regions are marked with green vertical bars, whose widths give the uncertainty in the regions. NH_3_ binding sites are marked with red vertical bars. NH_4_^+^ binding sites are marked Am1 (*z* = 1.07 nm) and Am5 (*z* = −1.37 nm), along with an additional binding feature, circled in blue (*z* = 1.32 nm). The bound state of NH_4_^+^ at each of the three sites is shown in molecular detail (labeled panels). All specific interactions within each site are described in the text.

To glean information about cation interaction with the channel interior, as well as specific binding interactions, we combined data from local *z*-dependent ion density profiles with umbrella sampling data (Materials and Methods) to calculate the potential of mean force (PMF) governing the transport of a single NH_4_^+^ molecule along the channel (*z-*) axis. The PMF profile, shown in Figures 3 and S3, details the free energy required to bring a single NH_4_^+^ molecule to any point within the channel from the bulk electrolyte on either side of the membrane. It clearly shows two free energy minima (binding sites)—one on either side of the membrane. While approaching either site from the bulk, the PMF indicates that NH_4_^+^ overcomes a very small barrier (∼2 *RT* on either side) at *z* ≈ ± 2nm (*z* = 0 nm is taken to be the center of mass of the trimer backbone).

The site on the periplasmic side (at *z* = 1.07 nm) of the membrane (labeled Am1 in Figure 3) is at precisely the location of site Am1 in the X-ray structures [17,18], although there are slight differences in the specific interactions. At this site, NH_4_^+^ forms hydrogen bonds with the backbone carbonyl oxygen of A162 and the sidechain hydroxyl oxygen of S219. The “other half” of the cation donates hydrogen bonds to a shell of water molecules, which is stabilized, in turn, by hydrogen bonds to the polar sidechain of Q104. Just below the cation, F107 provides a “floor” for the Am1 binding site.

We also note a feature in the free-energy profile (circled in blue at *z* = 1.342 nm) slightly above what is normally referred to as site Am1. This feature shows a slightly lower free energy than Am1, and corresponds to interaction with backbone carbonyl oxygens of D160, F161, and A162, which usher NH_4_^+^ into Am1. Figure 3 shows that the carboxylate of D160 accepts hydrogen bonds from the hydroxyl and amide nitrogen of T165, and from the backbone amide nitrogen from G164 and G163. These persistent interactions support D160′s structural role in orienting the carbonyl groups of the M4–M5 loop to recruit NH_4_^+^ from the periplasm. This is not to say that D160 does not stabilize the cation at the lower site, Am1, via through-space electrostatic interactions as suggested by Luzhkov et al. [22], but that its loop-stabilizing role (for recruitment of cations) is likely not separable from its electrostatic role. Nygaard et al. noticed in their simulations that NH_4_^+^ can occupy different substates (so-called Am1a or Am1b) within the Am1 binding site [23]. While, indeed, these substates may exist and contribute to the free-energy profile in Figure 3, the fact that the Am1 free-energy well is rather smooth implies that the free-energy barriers and differences between any set of substates would not be observable in a macroscopic (experimental) binding assay. In fact, the barrier (and free-energy difference) between Am1 and the feature circled in blue in Figure 3 is less than 1 *RT,* which implies that both features should be considered together in any discussion of ion recruitment and binding. Thus, it would make sense, from a free-energetic perspective, to refer to both of these “substates,” together, as the periplasmic binding site.

The PMF in Figure 3 also reveals a cation binding site on the cytoplasmic side of the membrane (labeled Am5 at *z* = −1.37 nm in Figure 3) whose existence was previously unknown according to the latest diffraction and computational studies. A NH_4_^+^ molecule at this new site, Am5, shows an equivalent level of stability to one bound at Am1, is mostly hydrated (usually by three to four water molecules), and donates strong hydrogen bonds to the carboxyl oxygen of D313 and the hydroxyl oxygen of S263.

A global view of the PMF for NH_4_^+^ translocation across the hydrophobic pore between the inner and outer vestibules reveals that NH_4_^+^ permeation would be extremely rare (Figure S3). The free energy barrier for NH_4_^+^ entering the lumen from either the periplasm or cytoplasm is prohibitive (>50 *RT* to reach the NH_3_ sites Am2, Am3, or Am4—see Figures 3 and S3). The free-energy barrier preventing complete translocation of NH_4_^+^ is astronomical (∼100 − 150 *RT,* see Figure S3), and is located near the center of the pore around the central NH_3_ binding site, Am3.

### Free-Energetic Analysis of NH_3_ Translocation

**Figure 4:**
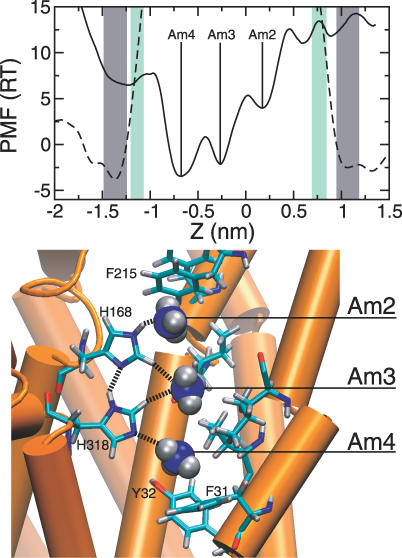
PMF for NH_3_ Along the Transport Axis In units of *RT* = 2.48 kJ/mol at 298 K (solid black line). The origin is the same as in Figure 2. Am (de)protonation regions are marked with green vertical bars, whose widths give the uncertainty in the regions, and NH_4_^+^ binding sites are marked with gray vertical bars. The PMF profile for NH_3_ (solid line) has been shifted such that its value coincides with that of NH_4_^+^ (dashed black line) at the equivalence ((de)protonation) regions (at the center of each green bar). Thus, the free-energy barrier for bringing NH_4_^+^ from site Am2 to the periplasmic equivalence point is ∼15 *RT* units. NH_3_ binding sites are marked Am2 (*z* = 0.17 nm), Am3 (*z* = −0.27 nm), and Am4 (*z* = −0.68 nm). The free-energy barrier for bringing NH_3_ (assuming single occupancy) from site Am4 to the cytoplasmic equivalence point is ∼10 *RT* units. The bound state of NH_3_ at each of the three sites is shown in molecular detail (bottom panel). All specific interactions within each site are described in the text.

We also calculated the PMF profile associated with translocation of a single NH_3_ molecule from the hydrated periplasmic vestibule to the cytoplasmic vestibule (see Figure 4). The profile produces three minima (sites) that were found in exactly the NH_3_ positions of Am2 (*z* = 0.17 nm), Am3 (*z* = −0.27 nm), and Am4 (*z* = −0.68 nm) from diffraction studies [17], thus we label them accordingly. Small “oscillations” in the profile are observed at *z* ≈ 0.4 − 1.0 nm corresponding to NH_3_ passage across the phenyl groups, F107 and F215, forming the “ceiling” of the pore. A similar feature is observed on the cytoplasmic end of the pore just after site Am4 at *z* ≈ −1.1 nm corresponding to NH_3_ passage across the phenyl side chain of F31, forming the “floor” of the hydrophobic pore (lumen). Residues H168 and H318 are seen to form stabilizing interactions with NH_3_ at each binding site (Figure 4, bottom). The most stable site at single NH_3_ occupancy is seen to be Am4, at −3.46 *RT* units. The barrier for bringing NH_3_ from Am4 to one of the NH_4_^+^ sites (Am1 or Am5) is drastically lower (∼9 − 16 *RT,* see Figure 4) than the barrier for bringing NH_4_^+^ to any one of the NH_3_ sites (Am2, Am3, or Am4, see Figure S3).

**Figure 5:**
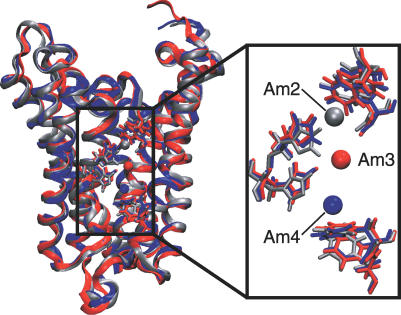
AmtB Is Structurally Invariant Regardless of Its Occupied State Structural alignment of channels occupied solely at site Am2 (silver), Am3 (red), and Am4 (blue). The window shows a closeup of the aligned binding sites. Slight differences are seen between each structure, but are within positional fluctuations.

The structure of the pore was seen to be structurally invariant regardless of whether a single NH_3_ molecule occupied Am2, Am3, or Am4 (Figure 5). Unlike other channels such as the K^+^ channel, whose conformation and activity is highly dependent upon the occupancy of permeant species [30,31], the AmtB channel architecture is stiff enough to maintain the structure of all three binding sites (Am1, Am2, and Am3), regardless of the occupancy of the pore. This makes our analysis of the pore convenient, because it means that the positions of the binding sites do not change even at higher occupancies. Thus, we may rely on a 1-D PMF (Figure 4) to enumerate the possible NH_3_ loading states.

### Apparent pK_a_ of NH_4_^+^ as a Function of the Transport Axis

**Figure 6:**
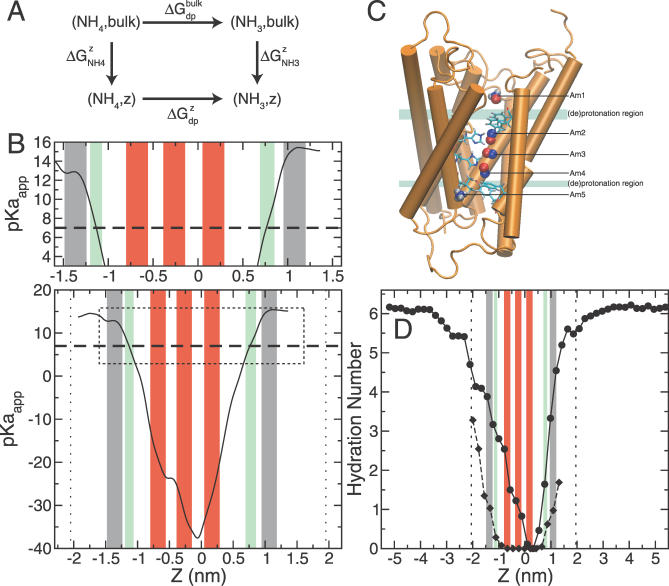
Apparent pK_a_ and Hydration of Am Along the Transport Axis (A) Free-energy cycle used to determine pK_a_ of NH_4_^+^ as a function of the transport axis.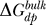 is the free energy for the alchemical reaction NH_4_^+^ → NH_3_ in the bulk, far from the membrane, and Δ*G* ^z^_dp_ is the free energy for the same reaction at a particular position, *z,* along the transport axis.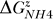 is the free energy to bring NH_4_^+^ from the bulk to a particular position, *z,* and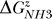 is the analogous quantity for NH_3_. (B) Apparent pK_a_ (pKa_app_) for NH_4_^+^ as a function of *z* for the relevant region of the channel. The top panel shows a closeup of the portion of the bottom panel enclosed in the rectangular box drawn with dashed lines. A large dashed black line marks neutral pH in both panels. Vertical dotted lines (bottom panel) mark the average positions of lipid phosphate groups. As in Figures 3 and 4, gray bars represent NH_4_^+^ binding sites, red bars represent NH_3_ binding sites, and green bars represent (de)protonation regions. The periplasmic (de)protonation region is at *z* = 0.77 ± 0.08 nm, and the cytoplasmic (de)protonation region is at *z* = −1.13 ± 0.07 nm. See the text for an explanation of the uncertainties (width of the green bars) in these regions. (C) Synopsis of binding sites and deprotonation regions generated by multiple structural alignment of AmtB channel structures singly bound at each binding site (a total of five structures taken from umbrella sampling simulations) and the X-ray structure [17]. Some transmembrane helices have been removed from the protein (orange) to allow visibility of the binding sites. The positions of the X-ray sites are shown as red spheres, and Am binding sites derived from our analysis are shown as either blue spheres (in the case of NH_3_) or blue spheres with white hydrogen atoms (in the case of NH_4_^+^). Any observed differences in the positions of the sites are within fluctuations. (De)protonation regions are marked in green. The periplasmic and cytoplasmic (de)protonation regions coincide with the phenyl groups of F107 and F31, respectively. (D) Hydration of NH_3_ (diamonds with dashed line) and NH_4_^+^ (circles with solid line) as a function of the transport axis derived by combining statistics from free MD production runs and umbrella sampling runs. The hydration number is defined as the average number of water molecules falling within the first coordination shell of Am. The first coordination shell of Am was taken to be the position of the first minimum in the N_Am_ − O_water_ pair correlation function (*R_coord_* = 0.362 nm, see Figure S4).

Since we know the free energy as a function of the transport axis in the case of either the protonated or unprotonated form of Am, it is possible to glean the apparent pK_a_ of NH_4_^+^ (*pK_a_*(*z*)) at all points along the transport pathway. This will be particularly useful in identifying precisely where its deprotonation occurs, and may even tell us how. The free-energy cycle in Figure 6A implies that we may calculate *pK_a_*(*z*) using Equation 1 (assuming *RT* units),

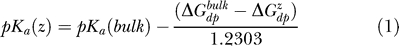
where pK_a_(bulk) is the pK_a_ of NH_4_^+^ in the bulk (known experimentally to have a value of 9.25), far away from the membrane surface, 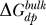 , is the free energy for the alchemical reaction NH_4_^+^ → NH_3_ in the bulk, far from the membrane, and 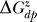 is the free energy for the same reaction at a particular position, *z,* along the transport axis (“*dp*” stands for “deprotonation”). We used the method of thermodynamic integration to obtain the necessary free-energy values (shown in Table 1) leading to the appropriate difference curve from the PMF profiles in Figures 3 and 4 (see Materials and Methods) yielding *pK_a_*(*z*).

**Table 1:**
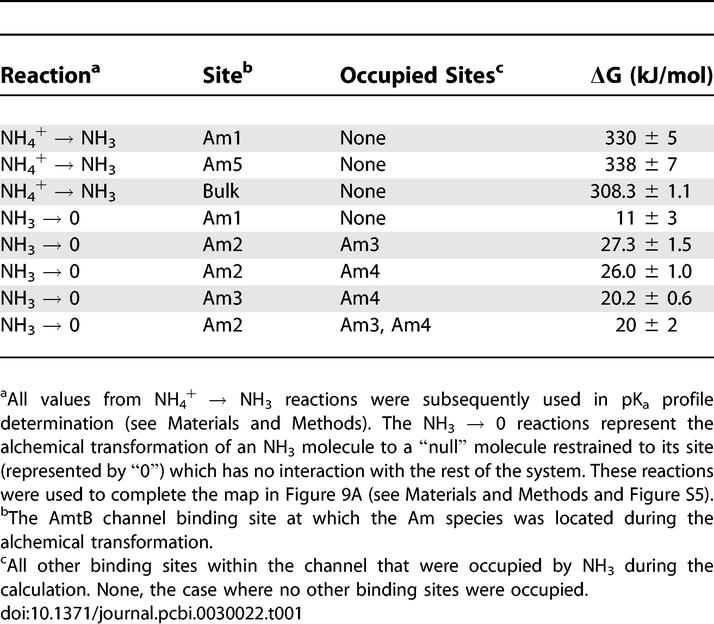
Gibbs Free Energy for Various Am Reactions Determined by Thermodynamic Integration

An explanation of the meaning of “apparent” pK_a_ (or −*log* K_a_) may be useful to the unacquainted reader. For a position, *z,* along the transport axis, the equilibrium constant for the proton dissociation event, NH_4_^+^ → NH_3_ + H^+^, is *K* = [*NH*_3_]_z_[*H* ^+^]_z_/[NH_4_^+^]*_z_*, where the square brackets denote populations (or concentrations). This quantity is not particularly useful experimentally (due to the ill-defined nature of the local pH or [H^+^]_z_) or in conventional MD simulations (due to the inherent design of water models, which represent only neutral pH). Thus, we turn to the “apparent” equilibrium constant, *K_a_* = [*NH*_3_]*_z_*[*H* ^+^]_bulk_/[NH_4_^+^]*_z_* to describe the favorability for Am to exist in its protonated or unprotonated form. This allows us to use the fixed value for pH for the bulk, which is well-represented by our force field, pH_bulk_ = 7. With this definition, a (de)protonation event will occur at the so-called “equivalence point,” when the apparent pK_a_ = pH_bulk_, or, in other words, when pK_a_ = 7. One must note that although, normally, the auto-ionization of water provides a range of possible values for the pK_a_ of 0–14 in aqueous solution, in a nonaqueous environment (say, in the absence of water or in an environment where water is nonbulk-like) this range does not apply.

The resulting NH_4_^+^ pK_a_ profile is shown in Figure 6B. Interestingly, the apparent pK_a_ is shifted upward by ∼5 units near the membrane surface and around sites Am1 and Am5. More precisely, using values from Table 1, the shift in pK_a_ is +4(±2) units at site Am1 (in agreement with a previous calculation [22]) and +5(±3) units at site Am5. This upward shift, disfavoring deprotonation, is not unexpected for several reasons. First, any region that stabilizes or binds NH_4_^+^ will necessarily foster the protonated form of Am. This is true, not only for the binding sites, Am1 and Am5, but also for the membrane surface itself (see Figures 2 and S2), which is seen to bind cations strongly—a result commonly seen at the membrane/electrolyte interface [26–29]. In addition, water has been shown to be highly polarized near the membrane surface and shows very different electrostatic properties than seen in the bulk [32]. The above arguments rationalize the upward shift in pK_a_ near the membrane surface and stabilization of Am in its protonated form at the Am1 and Am5 binding sites. Note that at some regions in the curve the apparent pK_a_ is seen to have values larger than 14, which violates limits implied by the auto-ionization of water. At these regions, 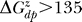 *RT* units, and deprotonation may be considered impossible. In addition, it is worth noting that the uncertainty accumulated in the thermodynamic integration calculations (Table 1) and in shifting the PMF profiles leads to an uncertainty in the pK_a_ shift of ∼3 units.

In the center of the hydrophobic pore containing sites Am2, Am3, and Am4 (Figure 6B, vertical red bars), we see that the pK_a_ of NH_4_^+^ becomes negative, again violating limits implied by water auto-ionization. In this region, 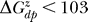 *RT* units, which implies that it is impossible for Am to exist in its protonated form. In fact, the pK_a_ profile indicates that Am must exist as NH_3_ well before reaching site Am2 on the periplasmic end, and well after leaving site Am4 on the cytoplasmic end of the pore (see Figure 6B, in between the vertical green bars). This result contrasts with qualitative analyses of static X-ray [17] or dynamic [23] channel structures, which posit that NH_4_^+^ can reach site Am2 before becoming deprotonated.

### The Periplasmic NH_4_^+^ Deprotonation Site

The equivalence point is where one would expect to find Am in the form of NH_3_ and NH_4_^+^ with equal probability, and is marked by the two green vertical bars in Figure 6B (we also show the bars in Figures 3 and 4). At neutral bulk pH, this is where deprotonation of NH_4_^+^ occurs. The width of each bar covers the range of certainty (∼3 pH units) for which the apparent pK_a_ is equal to the bulk pH, or, in our case, where *pK_a_*(*z*) = 7. Since the *pK_a_*(*z*) profile is extremely steep in this region, we may narrow down the deprotonation regions to very specific locations for both the periplasmic and cytoplasmic sides, despite the uncertainty in the pK_a_ profile.

On the periplasmic end of the channel, the site of deprotonation is immediately beneath site Am1 at *z* = 0.77 ± 0.08 nm (with respect to the center of mass of the trimer backbone). This site coincides exactly with the position of the phenyl side chain of F107 (see Figure 6C). As NH_4_^+^ travels from site Am1 toward the cytoplasm, its pK_a_ drops so steeply that it must become NH_3_ before passing the second phenyl group, F215. Based on Figure 4, the process of bringing NH_4_^+^ from site Am1 to the deprotonation region corresponds to crossing a free high-energy barrier of ∼15 *RT* units, which supports the experimental observation of very slow Am translocation rates of 10^5^–10^8^ ns/molecule [18].

If we observe the average hydration number of Am as a function of the transport axis (Figure 6D), we can rationalize the extremely unfavorable (15 *RT*) barrier to translocation and drastic drop in apparent pK_a_. In the bulk, NH_4_^+^ is hydrated by six water molecules, and at site Am1 (vertical gray bar at *z* ∼ 1.07 nm) it loses two to three water molecules from its hydration shell to form hydrogen bonds with A162 and S219 (Figure 3), but as the external electrochemical gradient pushes it toward the deprotonation site, its hydrogen bonds are stripped away such that eventually the only available acceptors are one to two water molecules, and the carbonyl oxygens of A162 and F215.

**Figure 7:**
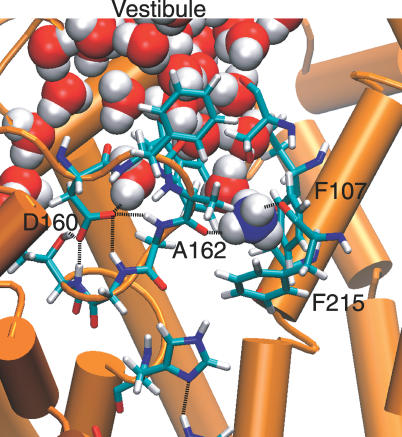
NH_4_^+^ at the Site of Deprotonation The configuration was taken from umbrella sampling simulations. NH_4_^+^ has full access to vestibular water, continuously connected to the bulk solution, to allow the escape of a proton to the bulk in the form of a hydronium ion.

The transition state for the NH_4_^+^ to NH_3_ transformation at the deprotonation site may be most accurately represented (given our classical molecular description) by Figure 7, where the F107 phenyl group is seen to allow passage by rotating in response to the presence of the cation. In this configuration, NH_4_^+^ is stripped to three hydrogen bonds with one water molecule and the carbonyl groups of A162 and F215, and must release one of its protons in order to favorably continue toward the cytoplasm as NH_3_. Previous works have suggested that the proton is donated to A162 [23] and, eventually, to D160 [20,23]. However, at this deprotonation site, NH_4_^+^ has access to periplasmic vestibular water, which connects to the bulk in a continuous fashion (Figures 6D and 7). Thus, it is much more likely to pass its proton directly to water, so that the proton may escape directly to the periplasm in the form of hydronium (cutting out the “middle man”). This conclusion is strongly supported by (1) the fact that water ionizes more easily than a carbonyl group; (2) the fact that at the transition state (Figure 7) the carboxylate of D160 is persistently engaged in hydrogen bonds with the hydroxyl and amide nitrogen of T165, and the amide nitrogens of G163 and G164; and (3) the finding that these strong interactions shift the pK_a_ of the D160 sidechain downward by ∼9 pH units, making its protonation effectively impossible [22]. Therefore, it appears that the role of D160 is not to accept a proton, but, as mentioned in structural analyses [17,18], to force recruitment of cations from the bulk by orienting periplasmic M4-M5 loop carbonyl groups, and by electrostatically stabilizing NH_4_^+^ at site Am1 [22]. We again emphasize that these roles for D160 are likely *not* mutually exclusive.

**Figure 8:**
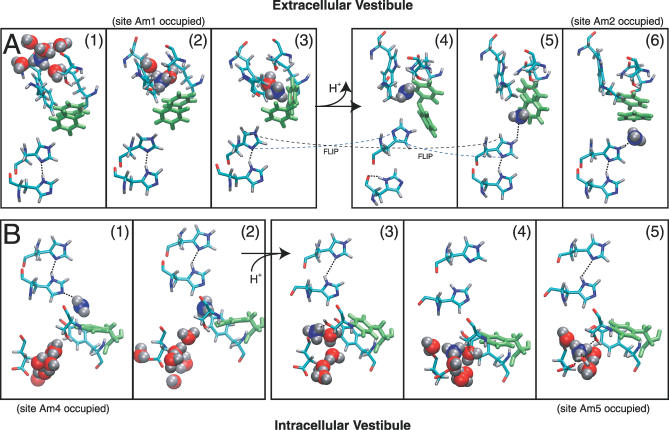
Am Deprotonation and Reprotonation Events reconstructed from snapshots taken from umbrella sampling simulations. Important Phe residues (F107, F215, and F31) are shown in light green. (A) Translocation of Am from site Am1 to site Am2. (A1) NH_4_^+^ in a fully hydrated state with six coordinating water molecules. (A2) NH_4_^+^ bound to site Am1. (A3) NH_4_^+^ overcomes an ∼15 *RT* free-energy barrier to reach the deprotonation region (also shown in Figure 7). F107 rotates to allow passage, and NH_4_^+^ hydration shell is stripped of all but one water molecule. (A4) After losing a proton, NH_3_ moves further toward site Am2, and all hydration is lost. F215 begins to rotate to allow passage. Here we notice a transient “flip” of the H168. This is only one of many conformational possibilities for the luminal His pair (H168 and H318). Different tautomeric states and conformations are also possible, but not represented here. (A5) F107 and F215 are both rotated and parallel, as NH_3_ accepts a hydrogen bond from H168, which has “flipped” back to assume its original hydrogen-bonded conformation with H318. (6) F107 and F215 are once again horizontal and parallel, as NH_3_ occupies site Am2. (B) Translocation of Am from site Am4 to site Am5. (B1) NH_3_ occupies site Am4, donating a hydrogen bond to H318. F31 and S263 interact with water, protecting the hydrophobic lumen from hydration. (B2) NH_3_ climbs an ∼10 *RT* barrier to approach the reprotonation region near F31, losing its hydrogen bond to H318, displacing F31, and moving toward water. (B3) After gaining a proton from water, Am proceeds as NH_4_^+^ and gains hydrogen bonds with water and S263 (hydroxyl oxygen). (B4) NH_4_^+^ becomes more hydrated as it moves toward site Am5. (B5) NH_4_^+^ binds to site Am5, and hydrogen bonds are formed with (three to four) water molecules, S263 hydroxyl oxygen, and D313 carboxylate oxygen.

It is also interesting to note that Figure 6D shows that the average hydration number of Am at the periplasmic deprotonation region (vertical green bar at 0.77 nm) drops immediately from 1–2 to 0 when NH_4_^+^ becomes NH_3_, as if this transition immediately allows Am to shed its final water to favorably enter the hydrophobic pore. In this sense, even though the reaction occurs in a condensed phase, it is much like the reaction that would occur when transferring Am from aqueous solution to a gas phase. The process of bringing Am from site Am1 (as NH_4_^+^) to site Am2 (as NH_3_) is summarized in Figure 8A.

### The Cytoplasmic NH_3_ Reprotonation Site

After NH_4_^+^ is dehydrated and deprotonated at the periplasmic deprotonation region (near F107), it continues as NH_3_, completely dehydrated (Figure 6D), and moves across sites Am2, Am3, and Am4. The consistent slope in the PMF (Figure 4), although all three NH_3_ binding sites provide stable sites, pushes NH_3_ toward the most stable site, Am4, on the periplasmic end of the hydrophobic lumen. Here, further passage is hindered by the phenyl group of F31. This sidechain, forming the “floor” of the hydrophobic lumen provides an ∼10 *RT* free-energy barrier (see Figure 4) for NH_3_ reaching the cytoplasmic (de)protonation region at *z* = −1.13 ± 0.07 nm (see the corresponding green bar in Figure 6B–6D). This region nearly coincides with the position of the phenyl group of F31 (Figure 6C).

The nature of the barrier to *re*protonate NH_3_ is seen to be similar in origin to that for *de*protonation of NH_4_^+^ near F107. Namely, it requires work to force NH_3_ toward the water-accessible cytoplasmic vestibule just beneath the F31 phenyl “floor” of the lumen. Presumably, larger NH_3_ luminal occupancy (i.e., more than 1 NH_3_) would lower the barrier to reprotonation due to mutual destabilization of NH_3_ molecules (we discuss this later). The protonation of NH_3_ to form NH_4_^+^ at the reprotonation region (green bar in Figure 6D) is coupled to a gain in hydration from 0 (coordinating NH_3_) to 2–3 (coordinating NH_4_^+^) water molecules and the gain of one hydrogen bond to the hydroxyl oxygen of S263 (see Figure 6D and Figure 8B3). Thus, again, the only plausible species involved in the protonation event is water. The process of bringing Am from site Am4 (as NH_3_) to site Am5 (as NH_4_^+^) is summarized in Figure 8B.

### Selective Permeability: The Role of Phenylalanine and the (Dehydrated) Hydrophobic Lumen

Given the results presented thus far, it is clear that phenyl side chains play a key role at both the periplasmic and cytoplasmic (de)protonation regions. At the periplasmic (de)protonation region, the presence of the phenyl sidechain from F107, though it rotates to allow passage, provides a constriction in the Am pathway such that most of its hydrogen bond acceptors (including water) are removed (Figure 7). Given the sequence of events observed in Figure 8A, it appears that once NH_4_^+^ donates a proton to neighboring water and moves slightly farther into the lumen, the F107 side chain guards against hydration while the second phenyl side chain from F215 rotates to allow passage of NH_3_. Together, F107 and F215 act as a “double doorway,” aiding to ensure dehydration of the passing Am species. In a similar manner, at the floor of the lumen, F31 guards against hydration from the cytoplasm (Figure 8B).

During 27.5 ns of equilibration and 25 ns of production MD with no intraluminal Am species, neither water nor NH_4_^+^ was seen to spontaneously enter the hydrophobic lumen (see Figure S4). During umbrella sampling simulations where NH_4_^+^ was forced to sample the lumen, it was seen to carry along anywhere from zero to three water molecules (compared with six in the bulk, see Figure 6D). However, as mentioned before, the free-energy barrier for NH_4_^+^ entering the lumen from either the periplasm or cytoplasm is prohibitive (>15 *RT* to reach the deprotonation region and >50 *RT* to reach the NH_3_ sites Am2, Am3, or Am4, see Figures 3 and S3). On the other hand, NH_3_ is quite “happy” to be completely dehydrated as it occupies the lumen (see Figures 4 and 6D). Therefore, we can conclude that the NH_4_^+^ ion's ability to deprotonate to form NH_3_ imparts Am with the ability to permeate the AmtB channel in a selective fashion. That is, permeation of Am or methylated Am will be greatly preferred over other aqueous inorganic ionic species because deprotonated Am (or methylated Am) can favorably occupy the hydrophobic lumen.

Based on our calculations, we can expect prohibitive free energy barriers (>50 *RT*) for *permanently* charged species to enter the lumen. Thus, without a doubt, this is the most robust rationale for the AmtB channel's display of selective permeability for Am over all *permanently* charged species (e.g., Family IA and IIA cations such as K^+^ or Na^+^), and is the most plausible source of the “Am-sensing” property of this channel [24]. With that being the case, the Phe residues, F107, F215, and F31, play a role in selective permeation, not by providing a larger relative stability at the periplasmic/cytoplasmic binding sites (Am1/Am5) for NH_4_^+^ over other cations, but by aiding in the removal of water at the deprotonation region of the transport pathway.

Whether other ions can competitively inhibit binding, and thus recruitment, of NH_4_^+^ to the periplasmic site, Am1, is a different question altogether (aside from selective permeability). However, this appears not to be an issue since experimental studies do not observe significant competitive inhibition of methyl-Am uptake by alkali cations in Amt/MEP/Rh transporters [6,33]. Recent calculations [22] reported slight selectivity of site Am1 for NH_4_^+^ over monovalent Family IA cations, rationalizing experimental observations of mild Am uptake inhibition. With only mild selectivity at site Am1, it appears that the hydrophobic lumen is the most Am-selective feature of the channel.

The finding that Am sheds its water before entering the hydrophobic lumen as NH_3_, where it remains dehydrated, contrasts with a recent computational study by Nygaard et al., which reported that the interior lumen of the hydrophobic pore is hydrated [23]. In that study, two 10-ns simulations, each with different neutral tautomeric states of the intraluminal His pair, H168 and H316, both demonstrated hydration of the lumen all the way up to the periplasmic phenyl “stack” provided by F107 and F215. The water entered the lumen in the form of a highly ordered, hydrogen bonded “water wire,” a very commonly observed arrangement in hydrophobic pores [34,35], which entered the pore via the cytoplasmic vestibule [23]. This observation led the authors to suggest that proton conduction by single-file water might provide a mechanism for H^+^ exchange with H168 and H316.

In contrast to the study of Nygaard, our MD simulations never displayed this hydration event in any one of the three channels in the AmtB trimer, despite 52.5 ns of dynamics (combining both equilibration and production runs). It is possible that a difference in choice of force field (see Materials and Methods) may have been the origin of these contrasting results. However, since the study of Nygaard et al. included only Cl^−^ counterions so as to achieve a net neutral system charge (no electrolyte) [23], we find it more probable that the main cause for our different observations is inclusion of NH_4_Cl electrolyte in our simulated system (measured to be 158 ± 5 mM in the bulk, see Figure 2B). We say this because past work has shown that simulated systems utilizing 3-D periodicity with full Ewald sums for treatment of electrostatic interactions and comprising membrane-bound proteins possessing a significant dipole moment (greater than ∼300 Debye) can display unexpected “water-ordering” artifacts [36,37]. This water-ordering phenomenon is constant in the bulk, can propagate all the way into the pores of membrane-bound channels, and is caused by the infinite array of persistent dipoles (caused by the membrane-bound protein in the central simulation cell) represented by a fully periodic system in 3-D. Normally, the largest dipole component of a membrane-bound protein will fall along the membrane normal (or transport axis). Thus, one way to remove the effect due to the electric field produced by the infinite, 3-D periodic dipolar array is to remove the periodicity in this direction (the membrane normal is usually taken to be the *z*-dimension), and to simulate in a slab (effectively 2-D or *xy*-) geometry [36]. There are other ways to avoid this artifact. For example, one can add electrolyte to the system (as we have done) so that it bears the dielectric response to the protein, rather than the water [37]. One may also choose to use a cutoff treatment of electrostatic interactions [37], although other artifacts are associated with this method. The addition of counterions alone, though it is important to assure a neutral system charge, does not appear to be enough to avoid the effect [36,37]. Nor does the addition of generous layers of water in bulk in an attempt to screen the effect [37].

The dipole moment of the AmtB trimer is several times larger (∼2,000 Debye, see Figure S1A) than the membrane proteins for which this phenomenon was first observed, and thus is an excellent candidate for providing an electric field to which the surrounding simulated system must respond. In our system, we observe that the dipole moment of the electrolyte precisely counteracts the dipole of the protein (Figure S1A), leaving a net zero dipole moment for the entire system (Figure S1B). Without the electrolyte, we can expect the water to bear the majority of the burden of the dielectric response to the protein.

The next obvious question is: Given that the net force of an electric field on a dipolar molecule is zero, why might this cause water to enter the hydrophobic lumen of AmtB? Recently, much attention has been given to the occupancy of water in hydrophobic spaces provided by macromolecular assemblies, including narrow hydrophobic pores, cavities, and the hydrophobic membrane interior [34,35,38,39]. It has been shown that, indeed, electric fields directed parallel to the axis of a narrow hydrophobic pore can make a water-filled state favorable, whereas in the absence of a field, it would be unfavorable [35]. In addition, electric fields directed along the bilayer normal can cause water to penetrate the hydrophobic portion of the lipid membrane itself and form water-filled pores—a process known as electroporation [38]. In either case, the electric field induces a preferred water orientation, which increases the probability of observing ordered water defects either at the membrane surface (in the case of membrane electroporation) or at the hydrophobic pore entrance (in the case of pore hydration). This appears to increase the probability that water enters the pore. It is also known that hydrophobic pore hydration occurs in an “all-or-none” fashion, well-represented by two-state models [34,35]. Once water enters a hydrophobic pore, we can expect that it will become filled. That is, one would assume, unless there is some feature that blocks full permeation. It would appear that the phenyl groups of F107 and F215 provide such a “blocking” feature.

### Free Energy and Dynamics of Multiply NH_3_-Occupied Luminal States

The three NH_3_ binding sites within the hydrophobic lumen proved to be highly disordered in the X-ray structure [17], leading to uncertainty in understanding whether the sites were occupied alternately with one another during the conductance mechanism, or partially occupied such that at high-bulk Am concentrations they might be fully occupied. According to the PMF for NH_3_ at single occupancy (Figure 4), it appears that immediately after the deprotonation event, NH_3_ is most likely to jump across sites Am2 and Am3 to site Am4. There it must either overcome a barrier (∼37 kJ/mol or ∼15 *RT*) to reprotonate and escape back to the periplasm, or overcome a barrier (∼25 kJ/mol or ∼10 *RT*) to reprotonate and escape to the cytoplasm. Multiple occupancy of the lumen will undoubtedly lower these barriers.

**Figure 9:**
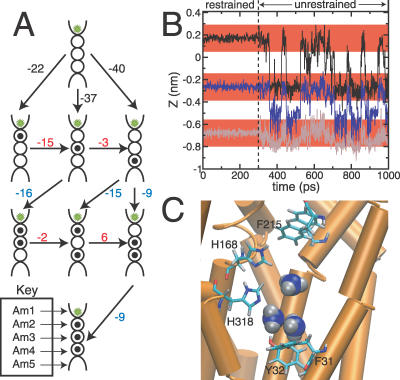
Dynamics and Favorability of Multiply NH_3_–Occupied Luminal States (A) State diagram of all possibly occupied states of the luminal NH_3_ sites. Each state is shown diagrammatically (from top to bottom, for a single state icon). (1) Site Am1 represented by a concave-up cusp with a green star, representing a reservoir of NH^3^. (2) sites Am2, Am3, and Am4 are represented by circles. (3) Am5 is represented by a concave-down cusp. Occupied (by NH_3_) luminal sites are represented by black dots (within the circles representing sites Am2–4). The free energy required for each reaction (arrows) is given in kJ/mol. Black values were determined from the PMF of Figure 4, red values were determined algebraically by completing the embedded thermodynamic cycles, and blue values were determined using thermodynamic integration techniques (see Figure S5 and surrounding discussion). (B) A demonstration of NH_3_ dynamics (1 ns) in the lumen. Horizontal red bars represent sites (listed in decreasing value of *z*) Am2, Am3, and Am4, respectively. For the first 300 ps of the simulation, the NH_3_ molecules are restrained (in the *z*-dimension only) to their binding positions. When the restraint is released, NH_3_ is seen to behave dynamically in the lumen, and there is a strong tendency for NH_3_ to move toward the site closest to the cytoplasmic end of the channel, site Am4. (C) A snapshot illustrating the tendency for NH_3_ to migrate toward site Am4. Here three NH_3_ molecules have migrated from a state where Am2, Am3, and Am4 are occupied, to a state where one molecule occupies Am3 and two molecules “fight” to occupy site Am4.

We utilized additional thermodynamic integration calculations (see Table 1 and Materials and Methods) to draw a state map for the possible NH_3_-occupied states of the lumen, shown in Figure 9A. For brevity, we adopt a vector representation for the occupied state of the three-site lumen, (O_2_,O_3_,O_4_), where O_2_, O_3_, and O_4_ are binary digits indicating whether Am2, Am3, or Am4, respectively, are occupied (O_i_ = 1) or unoccupied (O_i_ = 0). Since NH_3_ most favorably occupies site Am4 at single occupancy, (i.e., state 001), the map suggests that when a second NH_3_ enters, it will most likely occupy site Am2 (i.e., state 101). Figure 9A suggests that NH_3_ binding at site Am4 when site Am2 is occupied (the reaction 100 → 101) is 7 kJ/mol less-favorable than binding at site Am4 when no other site is occupied (the reaction 000 → 001). If we assume that the barrier for NH_3_ escape from Am4 to the cytoplasmic protonation region remains the same (we will refer to this as the “barrier assumption”) as calculated for single occupancy (Figure 4), then it would cost less free energy (∼18 kJ/mol or 7*RT*) for NH_3_ to move on to the cytoplasm from state 101. If NH_3_ were to dynamically move to a state where Am3 and Am4 are simultaneously occupied (state 011), we find that NH_3_ binding at site Am4 when site Am3 is occupied (010 → 011) is 28 kJ/mol less-favorable than binding at site Am4 when no other site is occupied (000 → 001). And with the above barrier assumption, the translocation of NH_3_ to the deprotonation region from site Am4 would become favorable, requiring ∼−3 kJ/mol or −*RT*. Needless to say, at full occupancy (state 111), the same approximation suggests that passage of NH_3_ from site Am4 to the deprotonation region would be even more favorable (∼−10 kJ/mol or −4*RT*).

Thus, our results suggest that doubly-occupied NH_3_ dynamics in the lumen can force the escape of NH_3_ at site Am4 to the cytoplasmic deprotonation region for successive binding to site Am5 and escape to the cytoplasm. The state most likely preceding the escape of NH_3_ to the deprotonation region is one where the lumen is occupied at sites Am3 and Am4 (state 011). We also note, however, that the free energy to attain full occupancy (the reaction 011 → 111) is favorable ∼−9 kJ/mol or −4 *RT*. In fact, this reaction is more favorable than the escape of NH_3_ to the cytoplasm. Therefore, it is also possible for the fully occupied state, 111, to precede escape of NH_3_ to the deprotonation region.

Though, above, we have only discussed the process of NH_3_ translocation toward the cytoplasm, our results do not preclude translocation toward the periplasm, which is well-known to occur [40]. In fact, at full occupancy (state 111), it becomes more favorable (by ∼−13 kJ/mol or −5 *RT*) for NH_3_ to escape from site Am2 to the periplasmic deprotonation region (i.e., the reaction 111 → 011) than the same reaction at zero occupancy (i.e., the reaction 100 → 000). Thus, our results are also consistent with the facilitation of gradient-dependent bidirectional NH_3_ transport.

In Figure 9B, we show a brief demonstration of NH_3_ dynamics for the fully occupied state of the lumen. Although the channel is fully occupied, it appears that the qualitative trend in binding-site favorability represented by the PMF of Figure 4 for single occupancy persists. The interactions of NH_3_ with the lumen display a strong tendency to force NH_3_ toward the cytoplasmic binding site, Am4. At times, the position of Am4 can be transiently occupied by two NH_3_ molecules (see Figure 9B and 9C). We can expect slight differences in the free energetics and dynamics of NH_3_ in the lumen depending on the tautomeric form of the intraluminal histidine residues [23]. The tautomeric configuration represented by our simulations appears to favor translocation toward the cytoplasm, while the study of Nygaard et al. showed that the alternate form for H168 and H318 does not appear to favor translocation in either direction. It is possible that, together, these different forms may be used by the channel to facilitate translocation toward the cytoplasm or toward the periplasm, depending on the electrochemical gradient conditions.

Thus, our results suggest that a periplasmic electrochemical gradient will push NH_4_^+^ from site Am1 to be deprotonated at the deprotonation region; the hydrophobic NH_3_ molecules begin to fill up the lumen, resulting in mutual destabilization or a buildup of “pressure,” which in turn allows NH_3_ reprotonation to become favorable. This means that the rate-limiting step of Am permeation is the NH_4_^+^ deprotonation step at the periplasmic end of the channel, representing an ∼15 *RT* free-energy barrier (see Figures 3 and 4) that must occur at the position of the F107 phenyl ring, before proceeding into the lumen, just underneath the F215 phenyl ring.

### Conclusions

Our results show that the global electrostatic nature of the AmtB channel inhibits anion binding to the periplasmic membrane and plays a large role in the recruitment of cations to the outer vestibule. PMF analysis verifies all crystallographically observed [17] Am binding sites and reveals a new cytoplasmic NH_4_^+^ binding site, Am5. At this new site, NH_4_^+^ donates hydrogen bonds to D313 and S263, as well as to 3–4 water molecules. Additional contributions near the periplasmic binding site, Am1, were found to be due to the backbone carbonyls of the M4–M5 loop. As mentioned in structural analyses [17,18], D160 plays an important structural role by ordering residues in the M4–M5 loop such that their backbone carbonyls may aid in recruiting periplasmic NH_4_^+^. This role persists as NH_4_^+^ binds to site Am1, and as it moves on to be deprotonated. The results of a recent study show that D160 stabilizes NH_4_^+^ bound to site Am1 [22]. This observation combined with our own results indicates that the structural role of D160 and its role in binding NH_4_^+^ are not mutually exclusive.

In contrast to other recent computational studies [20,23], our results do not support the hypothesis that D160 is involved in the deprotonation of NH_4_^+^. Nor does it support the hypothesis of structural analyses [17] that NH_4_^+^ deprotonation is mediated by intraluminal His residues (H168 and H318). Rather, our pK_a_ analysis of NH_4_^+^ shows that the deprotonation site must coincide with the position of the phenyl group of F107. This phenyl group rotates as NH_4_^+^ passes and is stripped of most of its hydrogen bonds. At this point, only the backbone carbonyl groups of A162 and F215 and a water molecule serve as hydrogen bond acceptors. Given (1) that water ionizes more easily than a carbonyl group, (2) that the carboxylate of D160 is persistently engaged in hydrogen bonds with residues of the M4-M5 loop, and (3) that the apparent pK_a_ of D160 is shifted downwardly by ∼9 pH units due to its stabilized interactions [22], water is the only plausible candidate for accepting a proton from NH_4_^+^ at the deprotonation site. The proton has full access to the periplasmic bulk, and must therefore escape in the form of hydronium. Thus, the Am deprotonation mechanism of AmtB is due to stripping NH_4_^+^ hydration to a critical point, much like the deprotonation event that one would observe in transferring Am from a hydrated state to a gas state.

Recently, a mutagenesis study showed that the twin luminal His residues (H168 and H318) are essential for substrate conductance [41]. Javelle et al. offered two explanations for this result: (1) these His residues serve as proton acceptors for entering Am such that they may move across the lumen as NH_3_, or (2) efficient conductance might require a “narrow mainly hydrophobic pore with a few precisely oriented hydrogen bond acceptor or donor functions for weak, stabilizing interactions with water/ammonia that still permit rapid diffusion.” Our results suggest that the latter explanation (2) applies.

The need of NH_4_^+^ for hydration, or some other form of hydrogen-bond stabilization, also manifests itself in our PMF analysis as an insurmountable free-energy barrier (>50 *RT*), preventing passage across the phenyl group of F107 and approaching any site within the lumen. This result contrasts with existing hypotheses [17,23] that NH_4_^+^ can occupy the intraluminal NH_3_ sites, Am2, or Am3. Since Am has the rare ability to deprotonate, it may enter the lumen as NH_3_. This is the origin of the Am-sensing ability of AmtB. Since most cations, such as Family IA and IIA ions, are permanently charged, their passage is expected to be disfavored, with a free-energy barrier of more than 50 *RT*.

Due to the nature of the deprotonation mechanism we describe, fully ordered hydration of the lumen would inhibit the Am-sensing ability of AmtB, providing the possibility for luminal Am to exist in its protonated form. Although, in contrast to other simulations of AmtB [23], the MD simulations of our work do not show any ordered luminal hydration (i.e., in the form of a water wire), they do not rule out the possibility of a few water molecules “sneaking” into the lumen. Such events would not be expected to inhibit NH_4_^+^ deprotonation (and, thus, AmtB's sensing capability).

Recent diffraction studies [17,41] clearly show electron density at the three luminal NH_3_ binding sites (as shown in Figure 4), but are unable to determine whether these sites are occupied by NH_3_ or water molecules. The diffraction study of Khademi et al. [17] could not find density corresponding to these sites for AmtB structures crystallized in the absence of 25mM AmSO_4_. This suggests that these peaks are mostly due to NH_3_ rather than water. Upon the introduction of charged species to the hydrophobic lumen, we might expect water to enter. For example, in the very unlikely event that NH_4_^+^ enters the lumen, we show that it can carry water with it (see Figure 6D). Recently, an X-ray diffraction study of an H168D mutant of AmtB showed electron density inside the lumen that might be attributable to water [41]. It was deemed most likely that this was due to the negative charge of the Asp side chain protruding into the lumen.

Given that hydration of narrow hydrophobic pores in the absence of an electric field is known to be unfavorable [35], the absence of ordered luminal hydration is most likely attributable to the presence of NH_4_Cl electrolyte in our simulated system, which, by responding to the macro-dipole of AmtB in the membrane, neutralizes the resulting electric field acting parallel to the transport axis. Similar “neutralizing” electrolytic responses to dipolar membrane proteins have also been observed in other work [37]. It appears that the periplasmic phenyl groups of F107 and F215 and the cytoplasmic phenyl group of F31 are major contributors to the maintenance of the dehydrated state of the AmtB lumen. This is most likely the reason why our NH_4_^+^ pK_a_ calculations highlight the positions of F107 and F31 as deprotonation “landmarks.”

Once Am occupies the lumen as NH_3_, in the singly occupied state, Am4 near the cytoplasm is the most stable site. As more hydrophobic NH_3_ is added to the lumen by means of NH_4_^+^ deprotonation from the cytoplasm, it may favorably become reprotonated from either a doubly occupied state (where Am3 and Am4 are simultaneously occupied) or a triply occupied state. Favorability for reaching the cytoplasmic reprotonation site is thus attained by a buildup of NH_3_ pressure in the lumen. In addition, the dynamics of NH_3_ within the lumen supports flow toward the floor of the lumen at the cytoplasmic site, Am4. Since, at the floor of the lumen, reprotonation of NH_3_ occurs favorably at multiply occupied states, the rate-limiting step for Am permeation must be the deprotonation step at the periplasm, where NH_4_^+^ becomes dehydrated. The free-energy barrier for this step is ∼15 *RT* based on our PMF calculations.

Though our study addresses mostly the translocation of Am toward the cytoplasm, the results we present are not inconsistent with the expected capability of bidirectional Am translocation. A previous study has found slight differences in the dynamics of NH_3_ in the lumen depending on the tautomeric form of the intraluminal histidine residues [23]. It is possible that, together, these different forms may be used by the channel to facilitate translocation toward the cytoplasm or toward the periplasm, depending on the electrochemical gradient.

Based on our apparent pK_a_ calculations, reprotonation of NH_3_ at the cytoplasmic end of the lumen was shown to occur upon hydration immediately after passing the phenyl group of F31. No plausible donor other than water exists at the point where NH_3_ reaches the reprotonation site. Thus, reprotonation at the cytoplasmic end of the channel must occur in the reverse manner to deprotonation at the periplasmic end. Upon reprotonation, Am binds as NH_4_^+^ to site Am5 before escaping to the cytoplasm. Thus, the role of the newly discovered site, Am5, is apparently to reduce the barrier to reprotonation by promoting hydration directly beneath F31 and by stabilizing NH_4_^+^ such that it does not escape back into the lumen as NH_3_.

## Materials and Methods

### Setup, equilibration, and production simulations of the AmtB system.

All MD simulations were performed using the GROMACS package [42,43], with the OPLS force field for protein, ions, and NH_3_ [44,45], the OPLS lipid parameters developed by Smondyrev and Berkowitz [46], and the SPC water model [47]. Our choice in force field parameters was guided by the fact that the OPLS force field, when used with the SPC water model, has been shown to yield equivalent (or better) results than the standard TIP3P water model when determining solvation free energies of amino acid side-chain analogs [48].

Production runs used a time step of 2.0 fs. Configurations of the system were saved every 1 ps for later analysis. Periodic boundary conditions were applied in all three dimensions. The LINCS algorithm was used to constrain all bonds in the systems [49]. Long-range electrostatics were handled using the PME algorithm [50], with a real-space cutoff of 0.9 nm. Other nonbonded interactions were truncated at 1.2 nm. The temperature was maintained at 298 K using the Nose–Hoover scheme with an oscillatory relaxation period of 2.0 ps. The pressure was maintained at 1.0 atm using the Parrinello–Rahman coupling scheme [51,52] with a barostat time constant of 2.0 ps. The rectangular simulation box was allowed to scale in size semi-isotropically to maintain pressure. Analysis of trajectories was performed using a combination of GROMACS analysis utilities and locally written scripts and programs.

The initial configuration of the AmtB trimer system was generated by first equilibrating a hydrated POPC bilayer containing 450 lipids (225 lipids per leaflet) and 29,889 water molecules for 4.0 ns. The area per lipid molecule was seen to converge to ∼0.62 nm^2^. The X-ray structure of Khademi et al. (PDB 1u7g) was used as the initial structure of the AmtB trimer in our simulations [17]. All residues were mutated such that a wild-type representation of the protein was obtained. In addition, the missing residues, A1 and P2, were added to each monomer to yield the full protein. All crystallographic water-molecule positions that coincided with the transmembrane portion of the trimer were stripped from the structure except for those deemed by Khademi et al. to be in “special positions.” We also removed NH_3_ positions from the interior binding sites (Am2, Am3, and Am4), to be replaced later for other calculations, leaving only the NH_4_^+^ position at site Am1 for each monomer. Hydrogen atoms were then added to the protein, NH_4_^+^ molecules, and crystallographic water molecules. All residues were assigned their standard (neutral pH) protonation states, and the tautomeric forms of each His residue were assigned using (geometric) hydrogen-bonding criteria except for the intraluminal H168 and H316 residues, which were shown to share a proton, and were thus assigned the tautomeric form suggested by Khademi et al. [17] and shown in Figure 4. The resulting structure used for subsequent equilibration contained three AmtB monomers, three NH_4_^+^ ions, and 486 water molecules.

A previous computational study [23] supported the tautomeric forms of H168 and H316 used in our simulations (shown in Figure 4) for the case where water is absent from the lumen. Since, for all of our simulations, water was not seen to enter the lumen, it would appear that our choice of tautomeric state is reasonable. It should be noted, however, that any conformational event observed involving these His residues during simulation (including umbrella sampling simulations) can only represent one of a few possibilities, since their protonation states must be permanently assigned. Of course, this is true of any simulation employing conventional force fields.

The trimeric structure described above was inserted into the equilibrated POPC bilayer, and all overlapping lipid and water molecules in the hydrated bilayer were removed. Water molecules in the bulk were randomly replaced by 117 Cl^−^ and 108 NH_4_^+^. The net charge on each AmtB monomer was 2*e,* thus with 117 Cl^−^ ions, and a total of 111 NH_4_^+^ ions (three coming from the X-ray structure), the net charge of the system was neutralized. The resulting initial system contained the AmtB trimer, 117 Cl^−^ ions, 111 NH_4_^+^ ions, 291 POPC molecules, and 28,099 water molecules (a total of 117,276 atomic sites).

**Figure 10:**
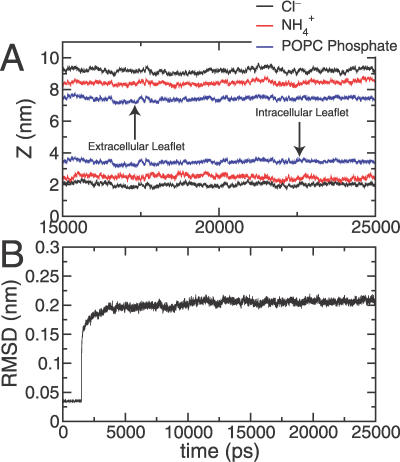
AmtB Trimer System Equilibration (A) Centers of mass of ionic species on each leaflet of the biomembrane as a function of time during equilibration. The center of mass of lipid phosphate is shown for reference. The origin in this plot is taken to be the corner of the simulation cell (as opposed to the center of mass of the AmtB trimer backbone as seen in other plots). (B) Root mean square deviation (RMSD) after alignment of AmtB protein structures from the equilibration trajectory with the initial structure of the simulation.

The system was minimized using the method of steepest descent, and simulated for 1.5 ns using Berendsen temperature and pressure coupling [53] while imposing restraints on the protein to allow membrane, ions, and water to relax around the protein. Restraints on the protein were then released, and the system was equilibrated further for 1 ns, again using Berendsen coupling. We then switched to Nose–Hoover temperature coupling and Parrinello–Rahman pressure coupling, and thermalized the system further, for 27.5 ns. We show the time evolution of the center of mass of both Cl^−^ and NH_4_^+^ for each leaflet of the biomembrane system (Figure 10A). It is seen that this quantity is well-converged after the first 15 ns of the equilibration run. The protein root mean square deviation (RMSD) was seen to converge after the first 15 ns of total simulation time as well (Figure 10B).

After the equilibration run, a 25-ns production run was performed. From this simulation, we derived the partial densities of various species across the simulation box (see Figures 2 and S2) and the hydration number of NH_4_^+^ across the simulation box (see Figures 6 and S4), to be used later, in conjunction with similar analyses of umbrella sampling trajectories. The average concentration of NH_4_Cl in the bulk as measured from the resulting partial densities was 158 ± 5 mM (see Figure 2B).

After the 25-ns production run, we placed three NH_3_ molecules into the luminal binding positions, determined from umbrella sampling simulations (Figure 4), within a single monomer of the final configuration. To demonstrate the dynamics of NH_3_ in the lumen, we performed 300 ps of simulation with the NH_3_ molecules harmonically restrained (in the *z*-dimension only—with a spring constant of 5,000 kJ/mol/nm^2^) to their binding positions, and 700 ps of simulation with the NH_3_ molecules unrestrained. The relevant data from these simulations is shown in Figure 9B.

### PMF calculations.

The final structure after 27.5 ns of equilibration was taken as the starting structure for umbrella sampling simulations [54]. All umbrella sampling windows utilized the same simulation conditions that were used for the production MD run. Am species (either NH_4_^+^ or NH_3_) were restrained to *z*-positions along the transport axis with respect to the AmtB trimer backbone (Am was free to move in the *xy*-plane). Each window utilized a harmonic umbrella potential acting in the *z*-dimension only, with a spring constant of 3,000 kJ/mol/nm^2^. Each umbrella simulation consisted of 150 ps of equilibration, followed by 150 ps of production MD over which statistics of Am occupancy along the transport axis (*z*) were recorded. Statistics from umbrella window simulations were combined and unbiased using the weighted histogram analysis method (WHAM) [55] to yield the probability, *P*(*z*), that a given Am species occupies position *z* along the transport axis. The PMF in units of *RT,* where *R* is the gas constant and *T* is the temperature (*RT* = 2.48 kJ/mol at 298 K), is then given by the Boltzmann relation *W*(*z*) = −ln*P*(*z*).

Given the size of the system and the large barriers required for translocation of the two Am species (i.e., involving displacement of Phe sidechains and (de)hydration barriers), we took a conservative, incremental approach to building the PMF for single NH_4_^+^ and NH_3_ translocation to ensure adequate relaxation of the system at each window simulation. In the first series of umbrella simulations, we sampled NH_4_^+^ along *z* from site Am1 (*z* = 1.04 nm), outwardly, into the periplasmic solution (*z* = 2.64 nm) using windows of Δ*z* = 0.04 nm (a total of 41 window simulations). Next, we sampled NH_4_^+^ along *z* from just beneath site Am1 (*z* = 1.00 nm), inwardly, into the lumen of the channel (down to *z* = −0.20 nm) using windows of Δ*z* = 0.04 nm (a total of 31 window simulations). In both series of simulations described above, the initial configuration of the (*i* + 1)th umbrella simulation was taken from the final configuration of the *i*th umbrella simulation. Thus, each simulation within a series was performed consecutively rather than in parallel (as is usually done) to ensure proper relaxation of the system along the Am pathway.

We then moved on to sample NH_3_ along its transport path starting within the channel lumen using two additional series of simulations. The initial configuration for these series was generated by removing the three Am1-bound NH_4_^+^ ions and three Cl^−^ ions from the bulk solution (to maintain net neutral system charge) in the initial structure of the NH_4_^+^ umbrella simulations described above. A single NH_3_ molecule was placed at site Am3 (the central luminal site, at *z* = 0.25 nm) within the AmtB monomer. We then sampled NH_3_ along *z* from site Am3 (*z* = 0.25 nm), outwardly, toward the periplasmic vestibule (up to *z* = 1.35 nm) using windows of Δ*z* = 0.04 nm (a total of 41 window simulations). Next, we sampled NH_3_ along *z* from just beneath site Am3 (*z* = −0.29 nm), inwardly, toward the cytoplasmic vestibule (down to *z* = −1.89 nm) using windows of Δ*z* = 0.04 nm (a total of 41 window simulations). Again, each simulation within a series was performed consecutively.

Finally, we sampled NH_4_^+^ translocation at the cytoplasmic end of the channel. The initial structure for these series of simulations was taken from the 27.5-ns equilibration run. The starting position of NH_4_^+^ was taken to be a position of NH_3_ near the phenyl group of F31 generated from umbrella sampling of NH_3_ translocation described above (at *z* = −1.10 nm). NH_4_^+^ translocation was sampled outwardly, toward the periplasm (up to *z* = −0.02 nm) using windows of Δ*z* = 0.04 nm (a total of 28 window simulations). We also performed an inward sampling series of simulations, starting from *z* = −1.14 nm toward the cytoplasm (down to *z* = −2.70 to nm) using windows of Δ*z* = 0.04 nm (a total of 40 window simulations).

After combining and unbiasing all NH_4_^+^ translocation umbrella statistics, we matched the resulting PMF at the periplasmic and cytoplasmic ends with the PMF derived from NH_4_^+^ densities provided by the 25-ns MD production run to extend the profile into the bulk region (Figure 3). The PMF was shifted such that its value in the bulk was zero. Both the NH_4_^+^ and NH_3_ PMFs were smoothed using window averaging. Taking into account all trajectories used in the construction of the PMFs in this work, including all umbrella simulations and the production run, a total of 91.6 ns were simulated. Production run configurations were saved every 1 ps, and statistics from umbrella sampling trajectories were taken every timestep, yielding an analysis of a total of 1.69 × 10^7^ configurations for constructing the PMFs. The total simulation time mentioned above (91.6 ns) does not take into account the equilibration run (27.5 ns), nor the thermodynamic integration calculations (eight calculations comprising 2.1 ns each—see the discussion below), which, when combined, give the total simulation time of this study: 135.9 ns.

### Alchemical transformations and pK_a_(z) determination.

We performed two types of free-energy calculations in this work (results summarized in Table 1): (1) to determine the free energy for mutating NH_4_^+^ to NH_3_ at particular locations along the transport axis, and (2) to determine the free energy for “turning off” all interactions between NH_3_ and the rest of the system at particular binding sites within the lumen of the AmtB channel. For each individual free energy calculation, the system topology was varied, using a coupling parameter, *λ,* from the initial state (*λ* = 0) to the final state (*λ* = 1). All transformations were performed over a set of 21 simulations, each of 100-ps length and carried out at different fixed values of *λ* (i.e., *λ* = {0.00,0.05,0.10,…,0.95,1.00}). Data from the final 90 ps of each simulation was split into nine blocks, each 10 ps in length, thus providing nine sets of data (21 values each for 〈*∂H*/*∂λ*〉*_P_*_,*T*_, where *H*(*λ*) is the hamiltonian of the system). These sets of data were integrated from 0 to 1 to obtain the free energy of the transformation,

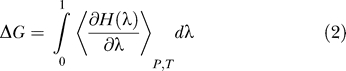
The nine resulting values for the free energy were averaged to obtain the final value, and an upper bound for the uncertainty in this value was taken to be the standard deviation of the sample.

The type 1 calculations were used in conjunction with the PMF profiles of Figures 3, 4, and S3 to determine the apparent pK_a_ as a function of the transport axis (Figure 6B). In these calculations, the NH_4_^+^ molecule (*λ* = 0) of interest was transformed to an NH_3_ molecule bonded to a dummy atom (*λ* = 1), where the dummy atom had no interactions with the remainder of the system. The pK_a_ calculation required (see Equation 1) the free-energy difference, 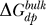 , for the NH_4_^+^ → NH_3_ transformation at some position in the bulk. For this transformation, we took *z* = 4.52 nm (note that in the bulk, all positions are equivalent by definition). In addition, we calculated the free energy, 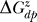 , for the same transformation at two different positions along the transport axis (*z* = 1.02 nm near site Am1, and *z* = −1.32 nm, near site Am5). This allowed us to shift the PMF profile for NH_3_ with respect to that of NH_4_^+^ (which was previously shifted to have a value of zero in the bulk) before subtracting one curve from the other to obtain 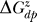 (see Equation 1).

Figure S3 shows two examples where the NH_3_ PMF profile was shifted according to the free-energy calculations. Figure S3A shows the result acquired when statistics from only one channel were combined for the derivation of the NH_4_^+^ PMF profile, and Figure S3B shows the result for the NH_4_^+^ PMF from umbrella sampling the translocation across two different AmtB monomers (and combining the data from both monomers). The uncertainty in 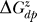 at sites Am1 and Am5 determined by thermodynamic integration had an upper bound in uncertainty of 7 kJ/mol (see Table 1). At these positions, this translates to an upper bound of uncertainty in the resulting pKa of 1.2 units. In addition, we note that the NH_3_ PMF profile could not be fit exactly to the points determined by thermodynamic integration (blue points with error bars in Figure S3). If, as an upper bound, we estimate the uncertainty to be 4 *RT* in the PMF profile, we obtain an additional uncertainty in the pK_a_ profile of 1.7 units. Combining these estimates of uncertainty in the pK_a_ calculation, we may give an upper bound in the determined pK_a_ profile (of Figures 6B and S3C) of 2.9 units (or ∼3 units, as mentioned in the text). The difference in the pK_a_ obtained when utilizing 1-channel versus 2-channel sampling (Figure S3) is obviously much greater than ∼3 units in the hydrophobic lumen of the channel (∼15–20 units around *z* = 0.1 nm, see Figure S3C); however, in this region, the total pK_a_ is about −45 units. Thus, regardless of the differences observed in this region, the apparent pK_a_ of NH_4_^+^ is so low that it would be effectively impossible for Am to exist in its charged form. Despite any differences seen in the results from 1-channel and 2-channel sampling, we come to the same conclusion when determining the position where deprotonation and reprotonation of Am occur along its pathway through the channel (green vertical bars in Figures 6B and S3C).

All type-2 calculations were used to complete the occupancy state map in Figure 9A (free-energy values shown in blue). In these calculations, the NH_3_ molecule (*λ* = 0) of interest at a particular site in the channel was transformed into a “null” molecule (*λ* = 1), where in the “null” state the NH_3_ molecule had no interactions with any other molecule within the system and was harmonically restrained to its site. The value of 〈*∂H*/*∂λ*〉*_P_*_,*T*_, at *λ* = 1, corresponding to the “null” state, was determined by linear extrapolation based on the tail-end values at *λ* = {0.85,0.90,0.95}. The free energy for each of these calculations is shown in Table 1. The free-energy values shown in the state map of Figure 9A were derived using the sequences of reactions shown in Figure S5B–S5E.

## Supporting Information

Figure S1 Time Series of System Dipole Moments The transport axis (*z*-) component is plotted.
(A) The AmtB trimer (black) shows a large, persistent dipole moment with very little fluctuation and an average value of 〈*μ_z_*〉 = −2040 ± 60 Debye. The dipole moment of all system ions combined (i.e., the NH_4_Cl electrolyte) is seen to counteract the dipole of the AmtB trimer with an average value of 〈*μ_z_*〉 = +2600 ± 1300 Debye. This value is precisely the same magnitude as the protein to within fluctuations due mostly to ion diffusion across the simulation cell boundary.
(B) The system dipole moment. The value is zero to within fluctuations (〈*μ_z_*〉 = 0 ± 1500 Debye), due to the dielectric response of the electrolyte.
(2.2 MB EPS)

Figure S2 Impact of AmtB versus POPC Membrane on System Ion Distributions Ion densities determined by calculating the average number of ions at a given position, *z,* that fall within 1.7 nm (in the *xy*-plane) of the center of mass of an AmtB monomer (black curves) and outside 3.7 nm (in the *xy*-plane) of the AmtB trimer center of mass (red curves).
(A) Conditional NH_4_^+^ densities. The red curve shows normal behavior for NH_4_^+^ binding to the membrane surface. On the black curve, the region from *z* ∼ 1–2 nm, near the periplasmic vestibule, shows a concentration ∼16 times larger than the region from *z* ∼ −1 to −2 nm, near the cytoplasmic vestibule.
(B) Conditional Cl^−^ densities. The red curve shows slightly less binding to the periplasmic membrane surface than the cytoplasmic surface. The black curve shows a concentration ∼3 times smaller near the periplasmic vestibule than the cytoplasmic vestibule.
(C) Illustration of the *xy*-planar regions for which the conditional density curves in (A) and (B) were calculated. The AmtB trimer is encircled by the gray cylinder with a radius of 3.7 nm whose center falls on the center of mass of the AmtB trimer. Three red cylinders of radius 1.7 nm encircle each AmtB monomer. Their centers coincide with the centers of mass of the three monomers.
(3.6 MB EPS)

Figure S3Analysis of the pK_a_ Profile for NH_4_^+^
(A) PMF for NH_3_ (red) and NH_4_^+^ (black) translocation as a function of the transport axis. The black curve is the result of umbrella sampling along *z* using a single monomer. The red curve was shifted with respect to the black curve based on thermodynamic integration calculations (datapoints shown in blue, see Table 1). A macroscopic view of the PMFs is shown in the inset. Note the large barrier for translocation (∼150 *RT*) of NH_4_^+^.(B) The same plot as shown in A, except the black curve is the result of sampling along *z* using two monomers (instead of only one). The major difference between the black curve here and that shown in A is the large barrier for NH_4_^+^ translocation (∼100 *RT,* see inset). In either case the barrier represents an impassable region for NH_4_^+^. Otherwise the curves are very similar.(C) The resulting apparent pK_a_ for NH_4_^+^ as a function of the transport axis when using data from 1-channel statistics, as in (A) (dotted line), and 2-channel statistics, as in (B) (dotted line), to determine 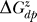 in Equation 1. The vertical colored bars have the same meaning as in Figure 6. pK_a_ = 7 is marked with a horizontal dashed line. Note that despite the differences in the relative PMF shifts, the pK_a_ profiles are nearly the same for all positive values, giving rise to precisely the same deprotonation region.
(1.0 MB EPS)

Figure S4 Hydration Number of NH_4_^+^ from Production MD (A) The pair correlation function between nitrogen of NH_4_^+^ and oxygen of H_2_O derived from simulation of our AmtB system. The first minimum, at *z* = 0.362 nm, is the first hydration shell boundary.
(B) Hydration of NH_4_^+^ from our 25-ns production run, calculated as the average number of water molecules falling within the hydration shell boundary of NH_4_^+^, at a given position. Hydration statistics were taken upon the condition that a NH_4_^+^ molecule was within 1.7 nm of the center of mass of a monomer backbone in the *xy*-plane (as was done in Figure S2 for partial-density calculations). Error bars were derived by dividing the trajectory into 500-ps blocks. Note that according to these data hydrated NH_4_^+^ does not pass the deprotonation regions at *z* = 0.77 ± 0.08 nm (periplasmic side) and *z* = −1.13 ± 0.07 nm (cytoplasmic side) from the aqueous solution to enter the channel. The statistics for this curve were combined with those derived from umbrella sampling trajectories to produce the NH_4_^+^ hydration curve in Figure 6D.
(533 KB EPS)

Figure S5 Calculation of Transition Free Energies in AmtB at Multiple Occupancy The diagrammatic representation for a given occupied state is described in Figure 9. A filled circle within a site represents NH_3_ occupancy, and an X within a site represents “null” occupancy (occupancy by a fictitious form of NH_3_ restrained to the site, and having no interactions with the system). Free energies for each reaction (in kJ/mol) are written above the yield arrows, and are shown with their uncertainties from thermodynamic integration calculations in Table 1. The free energies resulting from the reactions (B–E) were used in the state map shown in Figure 9A (blue free-energy values).
(A) The free energy to mutate NH_3_ to a null molecule at site Am1. This value was used as the initial step for reactions (B–E), using the assumption that NH_3_ at site Am1 is uncorrelated with the species in any luminal site.
(B–E) Various reactions necessary for filling in the state map (blue free-energy values) of Figure 9A. Note that the free energy to move null NH_3_ from one site to another is zero. All other free-energy values were derived from thermodynamic integration calculations (Table 1).
(672 KB EPS)

## Abbreviations

Am: ammonia or ammonium
Amt: ammonium transporters
MEP: methylamine permeases
POPC: palmitoyloleoylphosphatidylcholine
Rh: rhesus

## References

[pcbi-0030022-b001] Saier MHJR, Eng BH, Fard S, Garg J, Haggerty DA (1999). Phylogenetic characterization of novel transport protein families revealed by genome analyses. Biochim Biophys Acta.

[pcbi-0030022-b002] von Wirén N, Gazzarrini S, Gojon A, Frommer WB (2000). The molecular physiology of ammonium uptake and retrieval. Curr Opin Plant Biol.

[pcbi-0030022-b003] Ninnemann O, Jauniaux JC, Frommer WB (1994). Identification of a high affinity NH_4_
^+^ transporter from plants. EMBO J.

[pcbi-0030022-b004] Ludewig UN, von Wirén N, Frommer WB (2002). Uniport of NH_4_
^+^ by the root hair plasma membrane ammonium transporter LeAMT1;1. J Biol Chem.

[pcbi-0030022-b005] Sohlenkamp C, Wood CC, Roeb GW, Udvardi MK (2002). Characterization of *Arabidopsis* AtAMT2, a high-affinity ammonium transporter of the plasma membrane. Plant Physiol.

[pcbi-0030022-b006] Marini AM, Matassi G, Raynal V, André B (1997). A family of ammonium transporters in Saccharomyces cerevisiae. Mol Cell Biol.

[pcbi-0030022-b007] Ludewig UN, Wilken S, Wu B, Jost W, Obrdlik P (2003). Homo- and hetero-oligomerization of Ammonium Transporter-1 NH_4_
^+^ uniporters. J Biol Chem.

[pcbi-0030022-b008] Kleiner D (1985). Bacterial ammonium transport. FEMS Microbiol Rev.

[pcbi-0030022-b009] Siewe RM, Weil B, Burkovski A, Eikmanns BJ, Eikmanns M (1996). Functional and genetic characterization of the (methyl)ammonium uptake carrier of Cornebacterium glutamicum. J Biol Chem.

[pcbi-0030022-b010] Meier-Wagner J, Nolden L, Jakoby M, Siewe RM, Kramer R (2001). Multiplicity of ammonium uptake systems in Cornebacterium glutamicum: Role of Amt and AmtB. Microbiology.

[pcbi-0030022-b011] Le Van Kim C, Colin Y, Cartron J-P (2006). Rh proteins: Key structural and functional components of the red cell membrane. Blood Rev.

[pcbi-0030022-b012] Avent ND, Reid ME (2000). The Rh blood group system: A review. Blood.

[pcbi-0030022-b013] Knepper MA, Packer R, Good DW (1989). Ammonium transport in the kidney. Physiol Rev.

[pcbi-0030022-b014] Nakhoul NL, Hamm LL (2004). Non-erythroid Rh glycoproteins: A putative new family of mammalian ammonium transporters. Pfluegers Arch.

[pcbi-0030022-b015] Mayer M, Schaaf G, Mouro I, Lopez C, Colin Y (2006). Different transport mechanisms in plant and human AMT/Rh-type ammonium transporters. J Gen Physiol.

[pcbi-0030022-b016] Mayer M, Dynowski M, Ludewig UN (2006). Ammonium ion transport by the AMT/Rh homologue LeAMT1;1. Biochem J.

[pcbi-0030022-b017] Khademi S, O'Connell J, Remis J, Robles-Colmenares Y, Miercke LJW (2004). Mechanism of ammonia transport by Amt/MEP/Rh structure of AmtB at 1.35 Å. Science.

[pcbi-0030022-b018] Zheng L, Kostrewa D, Bernèche, Winkler FK, Li X-D (2004). The mechanism of ammonia transport based on the crystal structure of AmtB of Esterichia coli. Proc Natl Acad Sci U S A.

[pcbi-0030022-b019] Callebaut I, Dulin F, Bertrand O, Ripoche P, Mouro I (2006). Hydrophobic cluster analysis and modeling of the human Rh protein three-dimensional structures. Transfus Clin Biol.

[pcbi-0030022-b020] Lin Y, Cao Z, Mo Y (2006). Molecular dynamics simulations on the Escherichia coli ammonia channel protein AmtB: Mechanism of ammonia/ammonium transport. J Am Chem Soc.

[pcbi-0030022-b021] Liu Y, Hu X (2006). Molecular determinants for binding of ammonium ion in the ammonia transporter AmtB—A quantum chemical analysis. J Phys Chem A.

[pcbi-0030022-b022] Luzhkov VB, Almlöf M, Nervall M, Åqvist J (2006). Computational study of the binding affinity and selectivity of the bacterial ammonium transporter AmtB. Biochemstry.

[pcbi-0030022-b023] Nygaard TP, Rovira C, Peters GH, Jensen MØ (2006). Ammonium recruitment and ammonia transport by E. coli ammonia channel AmtB. Biophys J.

[pcbi-0030022-b024] Javelle A, Severi E, Thornton J, Merrick M (2004). Ammonium sensing in Escherichia coli. J Biol Chem.

[pcbi-0030022-b025] Marini AM, Boeckstaens M, Benjelloun F, Chérif-Zahar B, André B (2006). Structural involvement in substrate recognition of an essential aspartate residue conserved in Mep/Amt and Rh-type ammonium transporters. Curr Genet.

[pcbi-0030022-b026] Böckmann RA, Hac A, Heimberg T, Grubmüller H (2003). Effect of sodium chloride on a lipid bilayer. Biophys J.

[pcbi-0030022-b027] Mukhopadhyay P, Monticelli L, Tieleman DP (2004). Molecular dynamics simulation of a palmitoyl–oleoyl phosphatidylserine bilayer with Na^+^ counterions and NaCl. Biophys J.

[pcbi-0030022-b028] Pandit SA, Bostick D, Berkowitz ML (2003). Molecular dynamics simulation of a dipalmitoylphosphatidylcholine bilayer with NaCl. Biophys J.

[pcbi-0030022-b029] Pandit SA, Bostick DL, Berkowitz ML (2003). Mixed bilayer containing dipalmitoylphosphatidylcholine and dipalmitoylphosphatidylserine: Lipid complexation, ion binding, and electrostatics. Biophys J.

[pcbi-0030022-b030] Ray EC, Deutsch C (2006). A trapped intracellular cation modulates K^+^ channel recovery from slow inactivation. J Gen Physiol.

[pcbi-0030022-b031] Zhou Y, MacKinnon R (2003). The occupancy of ions in the K^+^ selectivity filter: Charge balance and coupling of ion binding to a protein conformational change underlie high conduction rates. J Mol Biol.

[pcbi-0030022-b032] Berkowitz ML, Bostick D, Pandit S (2006). Aqueous solutions next to phospholipid membrane surfaces: Insights from simulations. Chem Rev.

[pcbi-0030022-b033] Bakouh N, Benjelloun F, Hulin P, Brouillard F, Edelman A (2004). NH3 is involved in the NH4+ transport induced by the functional expression of the human Rh C glycoprotein. J Biol Chem.

[pcbi-0030022-b034] Beckstein O, Sansom MSP (2003). Liquid-vapor oscillations of water in hydrophobic nanopores. Proc Natl Acad Sci U S A.

[pcbi-0030022-b035] Vaitheeswaran S, Rasaiah JC, Hummer G (2004). Electric field and temperature effects on water in the narrow nonpolar pores of carbon nanotubes. J Chem Phys.

[pcbi-0030022-b036] Bostick D, Berkowitz ML (2003). The implementation of slab geometry for membrane-channel molecular dynamics simulations. Biophys J.

[pcbi-0030022-b037] Tieleman DP, Hess B, Sansom MSP (2002). Analysis and evaluation of channel models: Simulations of alamethicin. Biophys J.

[pcbi-0030022-b038] Tieleman DP (2004). The molecular basis of electroporation. BMC Biochemistry.

[pcbi-0030022-b039] Vaitheeswaran S, Yin H, Rasaiah JC, Hummer G (2004). Water clusters in nonpolar cavities. Proc Natl Acad Sci U S A.

[pcbi-0030022-b040] Soupene E, Lee H, Kustu S (2002). Ammonium/methylammonium transport (Amt) proteins facilitate diffusion of NH3 bidirectionally. Proc Natl Acad Sci U S A.

[pcbi-0030022-b041] Javelle A, Lupo D, Zheng L, Li X-D, Winkler FK (2006). An unusual twin–HIS arrangement in the pore of ammonia channels is essential for substrate conductance. J Biol Chem.

[pcbi-0030022-b042] Berendsen HJC, van der Spoel D, van Drunen R (1995). GROMACS: A message-passing parallel molecular dynamics implementation. Comput Phys Commun.

[pcbi-0030022-b043] Lindahl E, Hess B, van der Spoel D (2001). Gromacs 3.0: A package for molecular simulation and trajectory analysis. J Mol Modelling.

[pcbi-0030022-b044] Jorgensen WL, Maxwell DS, Tirado-Rives J (1996). Development and testing of the OPLS all-atom force field on conformational energetics and properties of organic liquids. J Am Chem Soc.

[pcbi-0030022-b045] Kaminski GA, Friesner RA, Tirado-Rives J, Jorgensen WL (2001). Evaluation and reparametrization of the OPLS-AA force field for proteins via comparison with accurate quantum chemical calculations on peptides. J Phys Chem B.

[pcbi-0030022-b046] Smondyrev AM, Berkowitz ML (1999). United atom force field for phospholipid membranes: Constant pressure molecular dynamics simulation of dipalmitoylphosphatidylcholine/water system. J Comput Chem.

[pcbi-0030022-b047] Hermans J, Berendsen HJC, van Gunsteren WF, Postma JPM (1984). A consistent empirical potential for water–protein interactions. Biopolymers.

[pcbi-0030022-b048] Shirts MR, Pande VS (2005). Solvation free energies of amino acid side chain analogs for common molecular mechanics water models. J Chem Phys.

[pcbi-0030022-b049] Hess B, Bekker H, Berendsen HJC, Fraaige JGEM (1997). LINCS: A linear constraint solver for molecular simulations. J Comput Chem.

[pcbi-0030022-b050] Essmann U, Perera L, Berkowitz ML, Darden T, Lee H (1995). A smooth particle mesh Ewald method. J Chem Phys.

[pcbi-0030022-b051] Nose S, Klein ML (1983). Constant pressure molecular dynamics for molecular systems. Mol Phys.

[pcbi-0030022-b052] Parrinello M, Rahman A (1981). Polymorphic transitions in single crystals: A new molecular dynamics method. J Appl Phys.

[pcbi-0030022-b053] Berendsen HJC, Postma JPM, van Gunsteren WF, DiNola A, Haak JR (1984). Molecular dynamics with coupling to an external bath. J Chem Phys.

[pcbi-0030022-b054] Torrie GM, Valleau JP (1977). Nonphysical sampling distributions in Monte Carlo free-energy estimation: Umbrella sampling. J Comput Phys.

[pcbi-0030022-b055] Kumar S, Rosenberg JM, Bouzida D, Swendsen RH, Kollman PA (1995). Multidimensional free-energy calculations using the weighted histogram analysis method. J Comput Chem.

